# Clinical Relevance of ‘Cap’ and ‘Track’ Development after Recent Small Subcortical Infarct

**DOI:** 10.1002/ana.27182

**Published:** 2025-01-17

**Authors:** Yajun Cheng, Carmen Arteaga‐Reyes, Una Clancy, Daniela Jaime Garcia, Maria Del C. Valdés Hernández, Michael J. Thrippleton, Michael S. Stringer, Gordon W. Blair, Stewart Wiseman, Francesca M. Chappell, Junfang Zhang, Xiaodi Liu, Angela C.C. Jochems, Susana Muñoz Maniega, Eleni Sakka, Mark E. Bastin, Rosalind Brown, Caroline M.J. Loos, Stephen D.J. Makin, Ming Liu, Bo Wu, Fergus N. Doubal, Joanna M. Wardlaw, MSS‐2, MSS‐3 and the INVESTIGATE‐SVDs Study Group, Ian Marshall, Olivia K.L. Hamilton, Ellen Backhouse, Will Hewins, Rachel Locherty, Emilie Sleight, Alasdair G. Morgan, Cameron Manning, Iona Hamilton, Gayle Barclay, Donna McIntyre, Charlotte Jardine, Dominic Job, David Perry, Tom MacGillivray, Charlene Hamid, Martin Dichgans, Anna Kopczak, Marco Duering, Benno Gesierich, Karin Waegemann, Michael Ingrisch, Salvatore Rudilosso, Ernest Chui, Esther Janssen, Robert J van Oostenbrugge, Julie Staals, Maud van Dinther, Danielle Kerkhofs, Walter H Backes, Geert Jan Biessels, Hilde van den Brink, Laurien P Onkenhout, Tine Arts, Stanley DT Pham, Jeroen Hendrikse, Jaco JMZ Zwanenburg, Jeroen CW Siero, Alastair JS Webb

**Affiliations:** ^1^ Department of Neurology West China Hospital, Sichuan University Chengdu China; ^2^ Centre for Clinical Brain Sciences, Edinburgh Imaging, UK Dementia Research Institute, University of Edinburgh Edinburgh UK; ^3^ Department of Neurology & Institute of Neurology Ruijin Hospital affiliated with Shanghai Jiao Tong University School of Medicine Shanghai China; ^4^ Division of Neurology, Department of Medicine LKS Faculty of Medicine, The University of Hong Kong Hong Kong China; ^5^ Department of Neurology Antwerp University Hospital and Research Group on Translational NeuroSciences, Faculty of Medicine and Health Sciences, University of Antwerp Antwerp Belgium; ^6^ Centre for Rural Health, Institute of Applied Health Sciences, University of Aberdeen Inverness UK

## Abstract

**Objective:**

After a recent small subcortical infarct (RSSI), some patients develop perilesional or remote hyperintensities (‘caps/tracks’) to the index infarct on T2/FLAIR MRI. However, their clinical relevance remains unclear. We investigated the clinicoradiological correlates of ‘caps/tracks’, and their impact on long‐term outcomes following RSSI.

**Methods:**

We identified participants with lacunar stroke and MRI‐confirmed RSSI from 3 prospective studies. At baseline, we collected risk factors, RSSI characteristics, small vessel disease (SVD) features, and microstructural integrity on diffusion imaging. Over 1‐year, we repeated MRI and recorded ‘caps/tracks’ blinded to other data. We evaluated predictors of ‘caps/tracks’, and their association with 1‐year functional (modified Rankin Scale score ≥2), mobility (Timed Up‐and‐Go), cognitive outcomes (Montreal Cognitive Assessment [MoCA] score <26), and recurrent cerebrovascular events (stroke/transient ischemic attack/incident infarct) using multivariable regression.

**Results:**

Among 185 participants, 93 (50.3%) developed ‘caps/tracks’ first detected at median 198 days after stroke. ‘Caps/tracks’ were independently predicted by baseline factors: larger RSSI, RSSI located in white matter, higher SVD score, and higher mean diffusivity in normal‐appearing white matter (odds ratio [OR] [95% confidence interval {CI}], 1.15 [1.07–1.25], 6.01 [2.80–13.57], 1.77 [1.31–2.44], 1.42 [1.01–2.03]). At 1 year, ‘cap/track’ formation was associated with worse functional outcome (OR: 3.17, 95% CI: 1.28–8.22), slower gait speed (β: 0.13, 95% CI: 0.01–0.25), and recurrent cerebrovascular events (hazard ratio [HR]: 2.05, 95% CI: 1.05–4.02), but not with cognitive impairment.

**Interpretation:**

‘Caps/tracks’ after RSSI are associated with worse clinical outcomes, and may reflect vulnerability to progressive SVD‐related injury. Reducing ‘caps/tracks’ may offer early efficacy markers in trials aiming to improve outcome after lacunar stroke. ANN NEUROL 2025;97:942–955

Clinical lacunar stroke syndromes, caused by recent small subcortical infarcts (RSSI), account for approximately 25% of all ischemic strokes.^1^ RSSI is a hallmark feature of cerebral small vessel disease (SVD).^2^ Despite their small lesion size, the long‐term prognosis after RSSI is far from benign, with an increased risk of stroke recurrence and cognitive impairment.^3^, ^4^, ^5^ A better understanding of the factors determining outcomes after RSSI is needed to identify high‐risk patients for targeted interventions.

Several clinical and imaging factors have been associated with adverse outcomes following RSSI, including clinical severity, infarct size, and neuroimaging markers of SVD.^5^, ^6^, ^7^ Additionally, there is growing interest in the long‐term morphological changes of RSSI.^2^ In some cases, the infarct may disappear, likely reflecting minimal tissue damage; while in others it may stay as white matter hyperintensity (WMH) or progress to cavitation (ie, more severe tissue damage), with cavitation associated with worse outcomes.^7^

Beyond these primary lesion changes, secondary perilesional and remote changes—referred to as ‘cap’ and/or ‘track’ formation—can occur over time (Fig 1).^8^, ^9^, ^10^ On MRI, these features appear as new hyperintensities extending upward (‘cap’) and/or downward (‘track’) from the RSSI tissue (hereinafter referred to as ‘caps/tracks’) and may indicate progressive degeneration of connected white matter tracts.^11^ Although the exact mechanisms are unclear, similar processes have been observed in subjects with cerebral autosomal dominant arteriopathy with subcortical infarcts and leukoencephalopathy (CADASIL), where WMH expansion and remote cortical thinning develop superficial to incident subcortical lacunes.^12^, ^13^, ^14^

**FIGURE 1:**
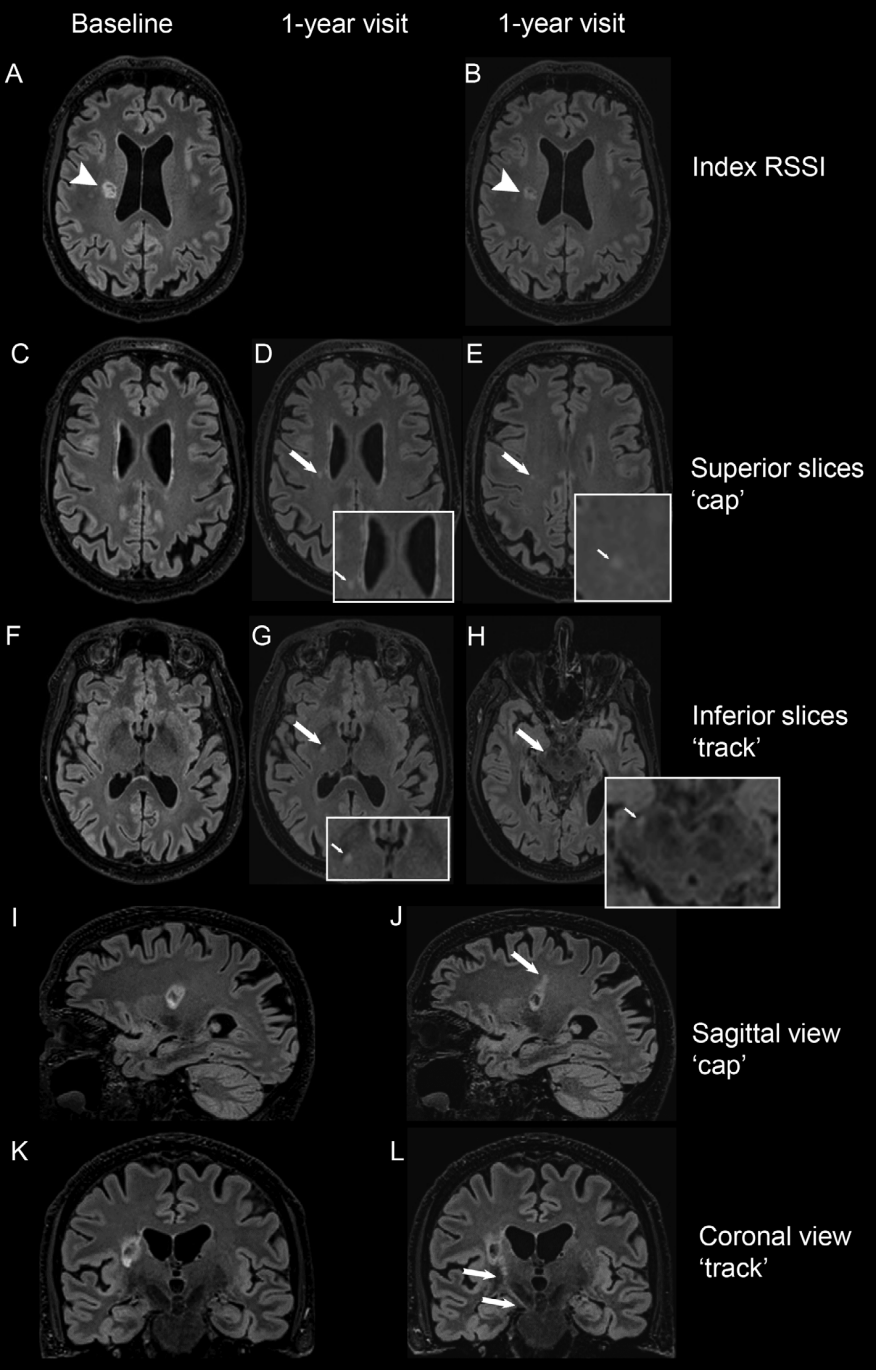
Example of ‘caps’ and ‘tracks’ following recent small subcortical infarct (RSSI) at the infero‐superior direction. The first column represents the baseline FLAIR MRI images performed 78 days after stroke onset, the second and third columns represent the 1‐year follow‐up FLAIR MRI images performed 476 days after stroke onset. (A, B) The index RSSI (arrowheads) is visible in the right centrum semiovale on baseline and 1‐year MRI. (C–E) On the MRI slice superior to the index RSSI, there is no abnormality at baseline. At 1 year, new ‘caps’ appear as hyperintensities in 2 axial slices superior to the index RSSI (arrows and augmented squares). (I, J) In the sagittal plane, the ‘cap’ is visible as a trajectory ascending from the index RSSI (arrow). (F–H) On the MRI slice inferior to the index RSSI, there is no abnormality at baseline. At 1 year, new ‘tracks’ appear as hyperintensities in 2 axial slices inferior to the index RSSI (arrows and augmented squares). (K, L) In the coronal plane, the ‘track’ is visible as a trajectory descending from the index RSSI toward the internal capsule and cerebral peduncle, following the corticospinal tract (arrows).

Our previous analysis of 79 patients with RSSI showed that half developed ‘caps/tracks’ during 1‐ to 5‐year follow‐up.^9^ The development of ‘caps/tracks’ was associated with baseline SVD markers like WMH and perivascular space (PVS) severity, but not with a specific vascular risk profile.^9^ However, these findings are based on unadjusted estimates and require validation in larger cohorts. Furthermore, the clinical relevance of ‘caps/tracks’ remains unknown.

Here, we aimed to use serial brain MRI to determine the frequency and timing of ‘caps/tracks’ during the year following RSSI, and their relationship with baseline vascular risk factors, stroke‐related features, SVD burden, and white matter microstructural integrity assessed with diffusion imaging (dMRI). In addition, we explored the association of ‘caps/tracks’ with 1‐year functional and cognitive outcomes, and recurrent cerebrovascular events. We hypothesized that clinical outcomes after lacunar stroke are influenced not only by the initial damage from the RSSI itself but also by the presence of secondary damage, as indicated by ‘caps/tracks’.

## Methods

This study follows the Strengthening the Reporting of Observational Studies in Epidemiology (STROBE) guidelines.^15^

### Participants

We identified participants with RSSI from 3 prospective observational cohort studies conducted in the UK. The Mild Stroke Study (MSS)‐2 (from 2010 to 2012, n = 264) and MSS‐3 (from 2018 to 2021, n = 229) recruited patients with minor ischemic lacunar or cortical stroke presenting to Edinburgh/Lothian stroke services. For the INVESTIGATE‐SVDs study (Imaging NeuroVascular, Endothelial & STructural InteGrity in prepAration to TrEat Small Vessel Diseases), we assessed patients with RSSI recruited in Edinburgh from 2017 to 2019 (n = 25). The MSS‐2 study was approved by the Lothian Research Ethics committee (ref 09/S1101/54). MSS‐3 and INVESTIGATE‐SVDs received approval from the Southeast Scotland Regional Ethics Committee (ref 18/SS/0044 and 16/SS/0123, respectively). All participants provided written informed consent. Details of the study protocols have been published.^16^, ^17^, ^18^

For the current analysis, inclusion criteria were: (1) A symptomatic lacunar syndrome with a compatible MRI‐defined RSSI. For MRI in the acute phase this was a recent infarction on diffusion‐weighted imaging (DWI), and for non‐acute MRI an anatomically relevant lacunar infarct visible on T2/fluid‐attenuated inversion recovery (FLAIR) and T1 sequences; and (2) availability of MRI scans at diagnosis and 1‐year follow‐up to observe the ‘caps/tracks’.

### Baseline Assessment

All participants underwent a thorough neurological examination by stroke physicians and a diagnostic MRI at presentation to stroke services. Stroke diagnosis was undertaken clinically by stroke experts and confirmed by brain imaging. Eligible patients were invited to attend the baseline study visit within 3 months after stroke. We recorded age, sex, education years, vascular risk factors (hypertension, diabetes mellitus, hyperlipidemia, smoking status), history of stroke or transient ischemic attack (TIA), ischemic heart disease, atrial fibrillation, blood pressure, and stroke severity (National Institutes of Health Stroke Scale, NIHSS). We assessed baseline cognition using Montreal Cognitive Assessment (MoCA), and premorbid intelligence using the National Adult Reading Test (NART).^19^

### Image Acquisition

In the MSS‐2 study, participants underwent a diagnostic MRI at presentation and a repeat MRI at baseline and 1 year, all on the same 1.5 T scanner (Signa LX; General Electric, Milwaukee, WI). The imaging protocols including dMRI, 2D T2‐weighted, FLAIR, gradient‐recalled echo, and 3D T1‐weighted sequences are published elsewhere.^16^, ^20^

In the MSS‐3 study, participants received diagnostic imaging, either at 3 T (Siemens Prisma as below) or 1.5 T (General Electric Signa HDxt as above) MRI. Participants underwent baseline MRI on a 3 T scanner (Prisma, Siemens Healthcare, Erlangen, Germany) with 3D isotropic T1‐weighted, 3D T2‐weighted, 3D FLAIR, dMRI, and susceptibility‐weighted imaging sequences. All participants had follow‐up MRI at 6 and 12 months, and a subset with moderate‐to‐high SVD burden had additional MRI scans at 3 or 9 months after baseline. All interval MRI from baseline to 1 year were performed on the same 3 T scanner and using the same sequences (full details described previously).^17^

In the INVESTIGATE‐SVDs study, participants had a diagnostic MRI at stroke onset and research MRI at variable times after stroke, using the same 3 T scanner and sequences as described for MSS‐3.^18^ For the current analysis, we included participants with available MRI at both diagnosis and 1 year.

Overall, participants from all 3 studies contributed to the analysis of ‘cap/track’ frequency at 1 year, while the MSS‐3 cohort, with its more frequent interval scanning, allowed a more detailed examination of the timeline for ‘cap/track’ formation.

### Image Analyses

**FIGURE 2:**
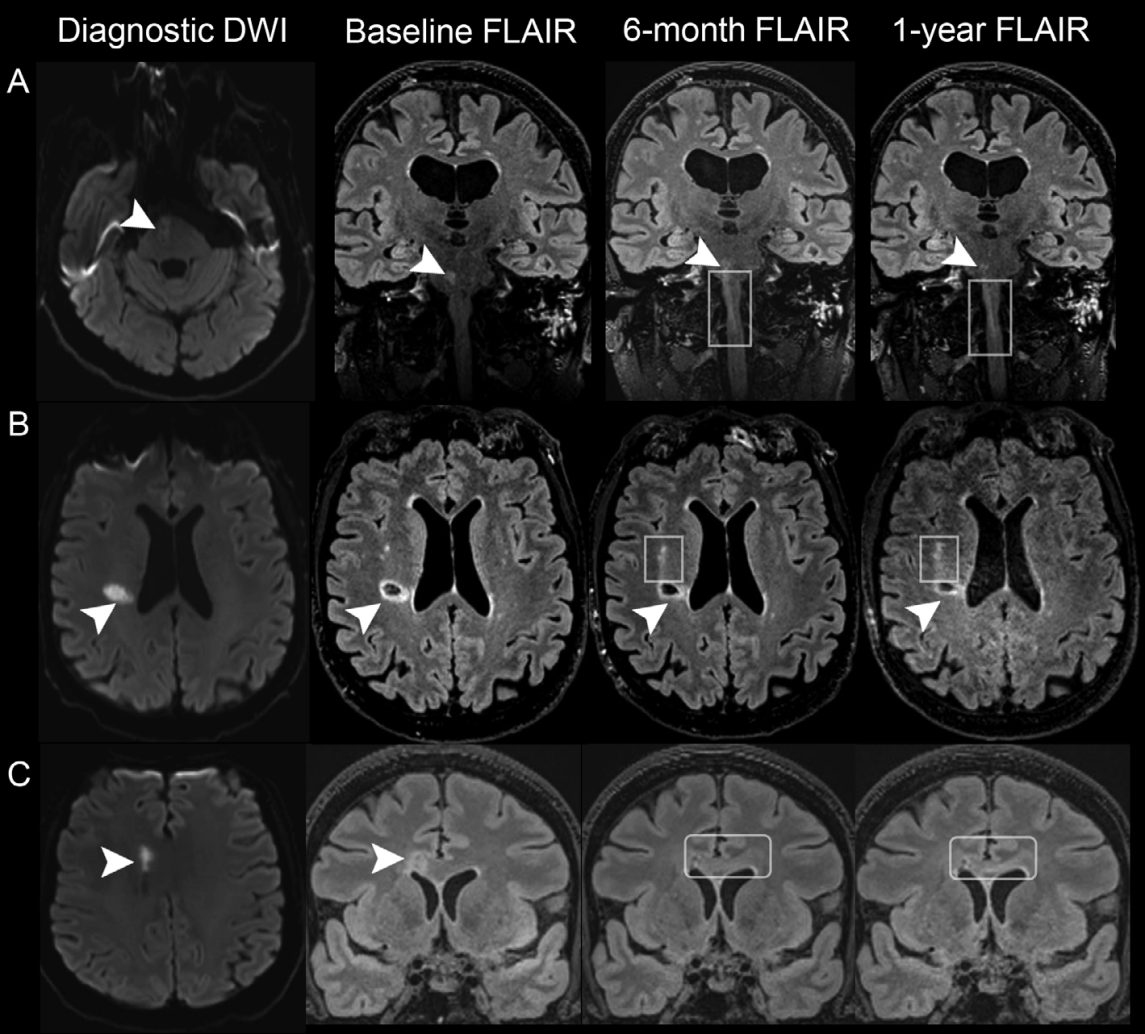
Different types of ‘tracks’ depending on the location of recent small subcortical infarct (RSSI). (Top row, A) In a patient with RSSI in the right pons (arrowheads), a ‘track’ develops and is visible on the 6‐month and 1‐year FLAIR scans as a trajectory descending from the index lesion toward the medulla (squares). (Middle row, B) In a patient with RSSI in the peripheral right coronal radiata (arrowheads), a ‘track’ develops and is visible on the 6‐month and 1‐year FLAIR scans, extending in the anterior intrahemispheric direction (squares). (Bottom row, C) In a patient with RSSI close to the corpus callosum (arrowheads), a ‘track’ develops and is visible on the 6‐month and 1‐year FLAIR scans. The ‘track’ traverses from the right to left hemisphere across the corpus callosum (squares). Note that the visibility of ‘tracks’ increases over time.

All image analyses were performed blinded to clinical data. We evaluated the development and characteristics of ‘caps/tracks’ based on Loos et al criteria^9^ and enhanced the clarity using 3D FLAIR, T2, and T1 imaging with co‐registration (Fig 1). All follow‐up scans were reviewed to record and categorize either ‘caps’ or ‘tracks’ based on their shape and position relative to the original infarct. Specifically, a ‘cap’ was defined as a new, round or ovoid‐shaped hyperintensity superior to the index RSSI, observed on any follow‐up scans subsequent to the diagnostic imaging. ‘Caps’ were more obvious on 3D‐T2/FLAIR images from coronal or sagittal views, with a trajectory extending superiorly from the RSSI. A ‘track’ was characterized as a new, rounded (often narrower than ‘cap’), elongated hyperintensity (visible ≥2 MRI slices), located inferior to the index RSSI on follow‐up scans. ‘Tracks’ were more evident on 3D‐T2/FLAIR images, with a trajectory extending inferiorly from RSSI along the descending white matter tract. The trajectory of ‘caps/tracks’ is variable depending on the RSSI location and affected white matter tracts. Besides the infero‐superior direction described above, some ‘tracks’ extended in the antero‐posterior intrahemispheric direction, particularly when the index RSSI was located in the peripheral deep white matter; or traversed to the contralateral hemisphere when the index RSSI was near the corpus callosum (Fig 2). Other sequences (DWI and T1) were used to rule out new or old infarcts and pre‐existing WMH in these regions. Two trained neurologists (Y.C. and C.A.R.) independently assessed ‘cap/track’ formation and any disagreement was adjudicated by an expert neuroradiologist (J.M.W.). Inter‐rater agreement was excellent for the presence of ‘caps/tracks’ (40 randomly selected scans, κ = 0.94).

We measured RSSI size in the x‐y (right‐to‐left, anterior‐to‐posterior), and z (superior‐to‐inferior) dimensions on the diagnostic FLAIR scan and reported the maximum diameter. The RSSI was categorized as mainly located in deep gray matter (lentiform nucleus, thalamus), white matter (internal or external capsule, centrum semiovale), or the brainstem. In case of multiple RSSI, we reported the one most likely to have caused the symptoms of the index stroke.

Baseline SVD markers were rated according to STRIVE criteria^2^ and graded using the Fazekas scale^21^ for WMH, PVS scale in basal ganglia and centrum semiovale (dichotomized into none‐to‐mild [degree 0–1] and moderate‐to‐severe [degree 2–4]),^22^ lacunes (number, location), and cerebral microbleeds using BOMBS scale.^23^ A summary SVD score was created as previously described.^24^ All images were reviewed by well‐trained raters and checked by J.M.W. WMH, normal‐appearing white matter (NAWM), and gray matter volumes were calculated and normalized for intracranial volume (ICV), using a validated semiautomated pipeline described previously.^17^, ^20^ To examine microstructural integrity, we fitted a diffusion tensor model using baseline dMRI scans to calculate fractional anisotropy (FA) and mean diffusivity (MD) in the NAWM.^25^

### Clinical Outcome Measures

All follow‐up visits were performed in person by trained researchers under the supervision of an expert consultant stroke physician, who was blinded to baseline clinical information and ‘cap/track’ assessment. Physical function, cognition, and recurrent cerebrovascular events were assessed.

Primary physical outcome was assessed at 1 year using modified Rankin Scale (mRS), with mRS ≥2 indicating functional impairment. Secondary physical outcomes included Timed Up‐and‐Go (TUG) test and Stroke Impact Scale (SIS). The TUG measures time (in seconds) to stand up from a chair, walk 3 meters, turn around, walk back, and sit down, with longer time reflecting slower gait speed and worse mobility. The SIS is a self‐reported stroke‐specific questionnaire covering 8 domains, with lower scores indicating worse impacts on life. We combined subdomains of strength, activities of daily living, mobility, and hand function into a SIS physical domain score.^26^ Note that TUG and SIS tests were performed at 3 years in the MSS‐2 study^3^ while all follow‐ups were at 1 year in the MSS‐3 and INVESTIGATE‐SVDs studies.^17^

Primary cognitive outcome was assessed using the MoCA score at 1 year, with <26 indicating cognitive impairment. Secondary cognitive outcome was the SIS memory/thinking domain score.

We combined recurrent stroke, TIA, or incident infarct on MRI during 1‐year follow‐up into a composite outcome of ‘recurrent cerebrovascular event’. Recurrent stroke or TIA syndrome were diagnosed either at stroke clinics or by the study team, adjudicated by a panel of stroke physicians and neuroradiologists. An incident infarct was defined as DWI‐positive lesion that appeared on follow‐up with or without apparent symptoms.^27^

### Statistical Analysis

Continuous variables were presented as mean (standard deviation [SD]) or median (interquartile range [IQR]) depending on data distributions; categorical variables were presented as count (percentage). Baseline characteristics of participants who did vs. did not develop ‘caps/tracks’ were compared using independent *t* test or Mann–Whitney *U* test for continuous variables, and χ^2^ test for categorical variables, as appropriate.

We used multivariable logistic regression models to investigate the association of ‘cap/track’ formation with clinical and imaging variables obtained at stroke diagnosis and baseline. To avoid multicollinearity, individual SVD features, summary SVD score and diffusion measures were included in separate models. Continuous variables without normal distribution (normalized WMH volume and TUG) were natural log‐transformed in the multivariable analysis. To avoid model overfitting, we calculated a vascular sum score (0–4), whereby 1 point was assigned for the presence of each of the following: hypertension, diabetes mellitus, hyperlipidemia, and current smoking.

We conducted multivariable logistic and linear regression models to investigate the association of ‘caps/tracks’ with physical and cognitive outcomes. Covariates were selected based on literature and clinical relevance. For physical outcomes, we adjusted for age, sex, NIHSS, summary SVD score, RSSI diameter, and recurrent cerebrovascular event. For cognitive outcomes, we adjusted for age, sex, education years, NART, baseline MoCA, and summary SVD score. We used Cox proportional hazards regression with time‐to‐recurrent cerebrovascular event (composite outcome) and for time‐to‐recurrent stroke/TIA, and logistic regression for MRI‐detected incident infarct by 1 year. Covariates included age, sex, vascular sum score, history of stroke/TIA, and summary SVD score. To account for potential clustering across the 3 source studies, we incorporated a study‐level variable into the models.^28^ The proportional hazards assumption was evaluated using graphical check and Schoenfeld residual–based tests. Effect sizes were reported as odds ratio (OR), hazard ratio (HR), standardized beta coefficient (β), and associated 95% confidence interval (CI). In a sensitivity analysis, we employed propensity score weighting to balance the distribution of observed confounders between the group with ‘caps/tracks’ and those without, and to examine the robustness of the associations with outcomes. A detailed methodological explanation of the propensity score weighting process is available in the Supplementary Methods.^29^

We further performed mediation analysis^30^ with the R package ‘mediation’ to test whether ‘caps/tracks’ explain the association between baseline summary SVD score and functional outcome that was previously reported.^31^ All statistical analyses were performed in R version 4.3.3 (R Foundation for Statistical Computing, Vienna, Austria). A *p* value <0.05 was considered statistically significant.

## Results

### Baseline Characteristics

**Figure 3:**
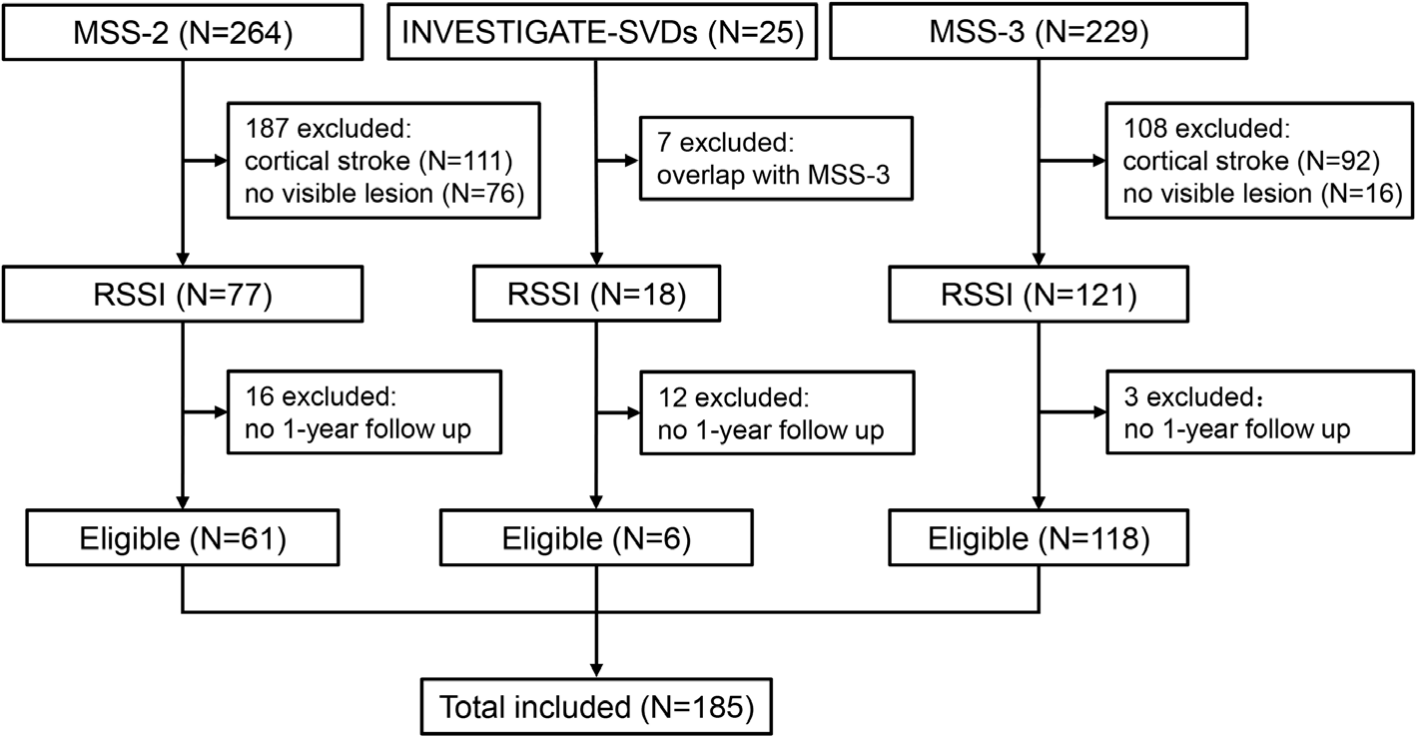
Flowchart of participant selection. RSSI, recent small subcortical infarct.

A total of 518 participants were screened from the 3 source studies. Of these, 302 were excluded due to non‐RSSI or participation in more than 1 of the source studies. A further 31 participants with RSSI were excluded due to absence of a 1‐year MRI. Thus, 185 RSSI participants were eligible in current analysis (Fig 3). Baseline characteristics are presented in Table 1 (see details stratified by source studies in Table S1). The mean age was 64.8 ± 11.2 years, 121 (65.4%) were men, and median NIHSS at presentation was 2 (IQR 1–3). Baseline characteristics were comparable between participants with and without 1‐year follow‐up, except that those who did not attend were older and less frequently had a history of hypertension (Table S2).

**Table 1:** Baseline characteristics of participants with or without ‘cap/track’ formation

Characteristics^a^	Overall (n = 185)	‘Cap/track’ present (n = 93)	‘Cap/track’ absent (n = 92)	*p*‐Value
**Demographics and risk factors**				
Age, year, mean (SD)	64.8 (11.2)	65.7 (10.3)	63.8 (12.0)	0.255
Male sex, n (%)	121 (65.4)	59 (63.4)	62 (67.4)	0.572
Education years, median [IQR]	11 [10–13]	11 [10–12]	11 [10–13]	0.497
Hypertension, n (%)	143 (77.3)	74 (79.6)	69 (75.0)	0.458
Diabetes mellitus, n (%)	38 (20.5)	21 (22.6)	17 (18.5)	0.490
Hyperlipidemia, n (%)	138 (74.6)	66 (71.0)	72 (78.3)	0.255
Current smoking, n (%)	56 (30.3)	28 (30.1)	28 (30.4)	0.961
Ischemic heart disease, n (%)	18 (9.7)	9 (9.7)	9 (9.8)	0.981
Atrial fibrillation, n (%)	13 (7.0)	7 (7.5)	6 (6.5)	0.789
History of stroke or TIA, n (%)	33 (17.8)	17 (18.3)	16 (17.4)	0.875
Vascular sum score, median [IQR]	2 [2–3]	2 [2–2]	2 [2–3]	0.809
**Clinical data**				
NIHSS, median [IQR]	2 [1–3]	2 [1–3]	2 [1–2.5]	0.400
MoCA, median [IQR]	25 [23–27]	25 [23–27]	24 [23–27]	0.767
NART, median [IQR]	36 [29–42]	36 [29–41]	35 [28–42]	0.813
Systolic blood pressure, mmHg, mean (SD)	150.5 (21.8)	152.3 (21.8)	148.8 (21.6)	0.275
Diastolic blood pressure, mmHg, mean (SD)	84.9 (13.5)	85.1 (14.6)	84.6 (12.2)	0.802
**RSSI diameter at diagnosis, mm, median [IQR]**	12.5 [8.3–15.2]	13.7 [11.0–16.4]	9.6 [6.0–14.2]	<0.001
**RSSI location, n (%)**				<0.001
Lentiform nucleus	13 (7.0)	7 (7.5)	6 (6.5)	
Internal or external capsule	35 (18.9)	22 (23.7)	13 (14.1)	
Thalamus	43 (23.2)	6 (6.5)	37 (40.2)	
Centrum semiovale	69 (37.3)	49 (52.7)	20 (21.7)	
Brainstem	25 (13.5)	9 (9.7)	16 (17.4)	
**Baseline SVD markers**				
Presence of lacunes, n (%)	107 (57.8)	60 (64.5)	47 (51.1)	0.064
No. of lacunes, median [IQR]	1 [0–3]	2 [0–5]	1 [0–2]	0.003
Periventricular WMH Fazekas score, median [IQR]	2 [1–3]	2 [1–3]	1 [1–2]	<0.001
Deep WMH Fazekas score, median [IQR]	1 [1–2]	2 [1–2]	1 [1–1]	<0.001
WMH volume as percent ICV, median [IQR]	0.8 [0.3–1.8]	1.1 [0.5–2.4]	0.5 [0.2–1.0]	<0.001
Moderate‐to‐severe BG‐PVS, n (%)	125 (67.6)	73 (78.5)	52 (56.5)	0.001
Moderate‐to‐severe CSO‐PVS, n (%)	139 (75.1)	75 (80.6)	64 (69.6)	0.081
Presence of cerebral microbleeds, n (%)	53 (28.6)	35 (37.6)	18 (19.6)	0.007
No. of cerebral microbleeds, median [IQR]	0 [0–1]	0 [0–2]	0 [0–0]	0.010
NAWM FA, median [IQR]	0.41 [0.27–0.43]	0.40 [0.26–0.42]	0.41 [0.28–0.43]	0.103
NAWM MD, 10^−3^ mm^2^/s, median [IQR]	0.77 [0.75–0.79]	0.77 [0.76–0.79]	0.76 [0.74–0.78]	0.009
Summary SVD score, median [IQR]	2 [1–3]	3 [2–4]	2 [1–2]	<0.001

BG‐PVS = basal ganglia perivascular spaces; CSO‐PVS = centrum semiovale perivascular spaces; FA = fractional anisotropy; ICV = intracranial volume; IQR = interquartile range; MD = mean diffusivity; MoCA = Montreal Cognitive Assessment; NART = National Adult Reading Test; NAWM = normal‐appearing white matter; NIHSS = National Institutes of Health Stroke Scale; RSSI = recent small subcortical infarct; SD = standard deviation; SVD = small vessel disease; TIA = transient ischemic attack; WMH = white matter hyperintensities.

^a^ The number of participants with missing values was 16 for education years, 46 for baseline MoCA, 18 for NART, 6 for WMH volume, and 13 for diffusion measures.

### ‘Cap/Track’ Frequency, Timing, and Predictors

**Figure 4:**
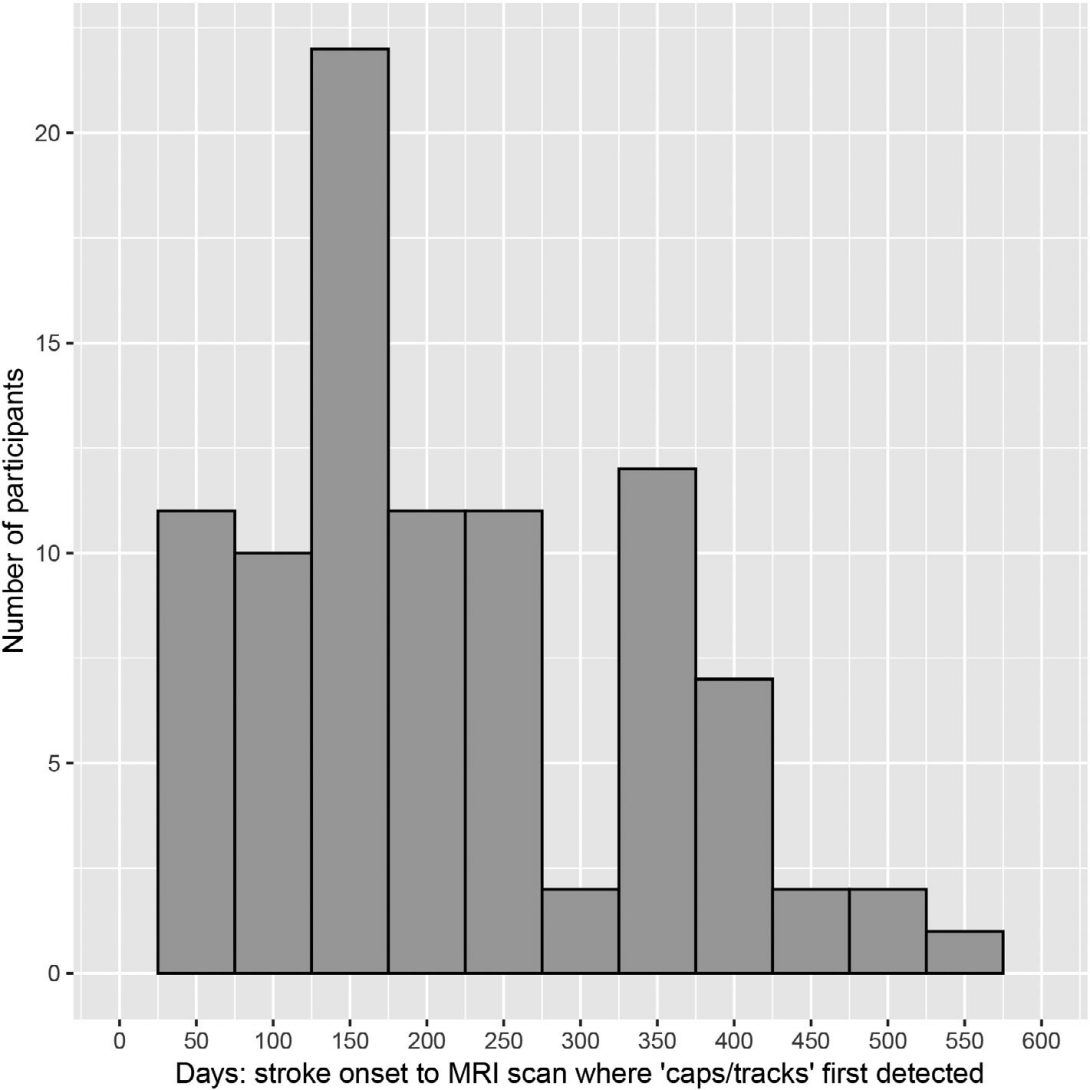
Distribution timeline of first detection of ‘caps/tracks’ on MRI across all visits.

The median interval between stroke onset and diagnostic imaging was 4 days (IQR 2–12), with baseline MRI performed at 53 days (IQR 37–70), and 1‐year MRI at 418 days (IQR 381–449). Additionally, 110 participants had MRI at 6‐month, 89 at 3‐month, and 30 at 9‐month after baseline. In the entire cohort, ‘caps/tracks’ were observed in 93 of 185 participants (50.3%), with similar frequencies between the MSS‐2 study (50.8%) and the MSS‐3/INVESTIGATE‐SVDs studies (50.0%). Specifically, 17 participants (18%) had ‘cap’ alone, 37 (40%) had ‘track’ alone, and 39 (42%) had both ‘cap’ and ‘track’ (Table S3). The median time from stroke onset to the MRI that recorded a new ‘cap/track’ was 198 days (IQR 135–354), with the highest frequency observed within 3‐ to 6‐months post‐stroke (Fig 4).

Compared to those without ‘cap/track’, participants with ‘caps/tracks’ had larger index RSSI, higher frequency of RSSI located in white matter, higher SVD burden, and higher NAWM‐MD on baseline MRI, whereas no difference was found in demographics or vascular risk factors between the 2 groups (Table 1). In the multivariable logistic regression model (Table 2), the development of ‘caps/tracks’ was associated with larger RSSI diameter (per 1 mm, OR: 1.15, 95% CI: 1.07 to 1.25), RSSI located in white matter (OR: 6.01, 95% CI: 2.80 to 13.57), higher lacune count (per 1 additional lacune, OR: 1.18, 95% CI: 1.06 to 1.35), larger normalized WMH volume (per ln unit increase, OR: 1.89, 95% CI: 1.31 to 2.78), moderate‐to‐severe basal ganglia PVS (OR: 2.47, 95% CI: 1.15 to 5.46), and higher summary SVD score (per 1 point, OR: 1.77, 95% CI: 1.31 to 2.44) at baseline. Regarding diffusion measures of white matter integrity, higher baseline NAWM‐MD (per 1 SD, OR: 1.42, 95% CI: 1.01 to 2.03) was associated with ‘cap/track’ formation, whereas no association was found for NAWM‐FA (per 1 SD, OR: 0.94, 95% CI: 0.66 to 1.33).

**Table 2:** Binary logistic regression analysis to identify baseline factors associated with ‘cap/track’ formation

Parameter	Univariable	*p‐*Value	Multivariable^a^	*p‐*Value
	OR (95% CI)		OR (95% CI)	
Age	1.02 (0.99–1.04)	0.254	1.02 (0.99–1.05)	0.196
Male sex	0.84 (0.46–1.54)	0.572	0.75 (0.35–1.57)	0.446
Vascular sum score	1.02 (0.72–1.43)	0.933	1.11 (0.75–1.66)	0.589
RSSI diameter, per 1 mm	1.17 (1.10–1.26)	<0.001	1.15 (1.07–1.25)	<0.001
RSSI location				
Deep gray matter	Reference		Reference	
White matter	5.40 (2.70–11.39)	<0.001	6.01 (2.80–13.57)	<0.001
Brainstem	1.86 (0.66–5.20)	0.235	2.83 (0.91–8.90)	0.071
No. of lacunes	1.17 (1.06–1.32)	0.004	1.18 (1.06–1.35)	0.006
Periventricular WMH Fazekas score	2.41 (1.67–3.58)	<0.001	2.27 (1.44–3.69)	0.001
Deep WMH Fazekas score	2.55 (1.69–4.01)	<0.001	2.36 (1.41–4.09)	0.001
Normalized WMH volume, per natural logarithm unit	1.90 (1.40–2.57)	<0.001	1.89 (1.31–2.78)	0.001
Moderate‐to‐severe BG‐PVS	2.81 (1.49–5.42)	0.002	2.47 (1.15–5.46)	0.022
Moderate‐to‐severe CSO‐PVS	1.82 (0.93–3.65)	0.083	1.20 (0.55–2.62)	0.639
No. of cerebral microbleeds	1.03 (0.99–1.08)	0.210	1.02 (0.98–1.08)	0.408
NAWM FA, per 1 SD	0.86 (0.63–1.16)	0.313	0.94 (0.66–1.33)	0.718
NAWM MD, per 1 SD	1.44 (1.06–1.99)	0.022	1.42 (1.01–2.03)	0.047
Summary SVD score	1.83 (1.43–2.40)	<0.001	1.77 (1.31–2.44)	<0.001

BG‐PVS = basal ganglia perivascular spaces; CSO‐PVS = centrum semiovale perivascular spaces; CI = confidence interval; FA = fractional anisotropy; MD = mean diffusivity; NAWM = normal‐appearing white matter; OR = odds ratio; RSSI = recent small subcortical infarct; SD = standard deviation; SVD = small vessel disease; WMH = white matter hyperintensities.

^a^ The multivariable model adjusted for age, sex, RSSI diameter, and RSSI location. Vascular sum score, individual SVD markers, diffusion measures and summary SVD score were entered the multivariable model separately.

### Association between ‘Cap/Track’ and Clinical Outcomes

All participants completed the 1‐year follow‐up for mRS, with 48 (25.9%) demonstrating functional impairment (mRS ≥2). Additionally, 145 (78.4%) underwent TUG and 162 (87.6%) completed the SIS evaluation. The development of ‘caps/tracks’ was associated with a higher likelihood of functional impairment (OR: 3.17, 95% CI: 1.28 to 8.22), slower gait speed (β for log‐transformed TUG: 0.13, 95% CI: 0.01 to 0.25), and lower SIS physical domain score (β: −6.97, 95% CI: −13.25 to −0.69) (Table 3).

**Table 3:** Multivariable regression analyses of associations between ‘cap/track’ and clinical outcomes

Parameter	‘Cap/track’ present (n = 93)	‘Cap/track’ absent (n = 92)	Effect size (95% CI)^a^	*p‐*Value
Physical function^b^				
mRS ≥2	35 (37.6%)	13 (14.1%)	OR: 3.17 (1.28–8.22)	0.014
TUG	11.2 [9.2–15.8]	10.0 [8.9–11.9]	β: 0.13 (0.01–0.25)	0.037
SIS physical domain score	79.5 (23.4)	88.0 (13.9)	β: −6.97 (−13.25– −0.69)	0.031
Cognition^c^				
MoCA <26	29 (34.9%)	32 (40.5%)	OR: 0.88 (0.36–2.16)	0.783
SIS memory/thinking domain score	83.1 (20.2)	84.0 (16.7)	β: −2.64 (−9.42–4.14)	0.447
Recurrent cerebrovascular event^d^				
Composite outcome	35 (37.6%)	15 (16.3%)	HR: 2.05 (1.05–4.02)	0.037
Recurrent stroke or TIA	12 (12.9%)	6 (6.5%)	HR: 6.62 (1.99–22.07)	0.002
Incident infarct on MRI	33 (35.5%)	11 (12.0%)	OR: 3.55 (1.50–8.98)	0.005

Data were represented as n (%), mean (SD), or median [IQR].

CI = confidence interval; HR = hazard ratio; MoCA = Montreal Cognitive Assessment; mRS = modified Rankin Scale; NART = National Adult Reading Test; NIHSS = National Institutes of Health Stroke Scale; OR = odds ratio; RSSI = recent small subcortical infarct; SIS = Stroke Impact Scale; SVD = small vessel disease; TIA = transient ischemic attack; TUG = Timed Up‐and‐Go.

^a^ The OR was estimated using binary logistic regression, the standardized beta coefficient (β) using linear regression, and the HR using Cox proportional hazards regression.

^b^ Adjusted for age, sex, NIHSS, baseline summary SVD score, RSSI diameter, recurrent cerebrovascular event, and source study level.

^c^ Adjusted for age, sex, education year, NART, baseline MoCA, baseline summary SVD score, and source study level.

^d^ Adjusted for age, sex, vascular sum score, history of stroke/TIA, baseline summary SVD score, and source study level.

In terms of cognitive outcomes at 1 year, 162 (87.6%) participants underwent MoCA and SIS assessment. Development of ‘caps/tracks’ did not show any association with cognitive impairment (OR for MoCA <26: 0.88, 95% CI: 0.36 to 2.16; β for SIS memory/thinking domain score: −2.64, 95% CI: −9.42 to 4.14) (Table 3).

During the study period, 50 participants (27%) experienced a recurrent cerebrovascular event. Of them, 18 (9.7%) had recurrent stroke or TIA, and 44 (23.8%) had incident infarcts on MRI, most of which were small subcortical infarcts (Table S4). The development of ‘caps/tracks’ was associated with increased risk of any recurrent cerebrovascular events (HR: 2.05, 95% CI: 1.05 to 4.02), recurrent stroke or TIA (HR: 6.62, 95% CI: 1.99 to 22.07), and MRI‐detected incident infarcts (OR: 3.55, 95% CI: 1.50 to 8.98) (Table 3).

Notably, outcomes worsened with increasing ‘cap/track’ formation, with participants exhibiting both ‘cap’ and ‘track’ presenting the poorest physical outcomes and highest risk of recurrent cerebrovascular events (Table S3). Similar results were found in sensitivity analyses with propensity score weighting (Table S5).

### Mediation Analysis of the Relationship among SVD Burden, ‘Cap/Track’ and Functional Outcome

**Figure 5:**
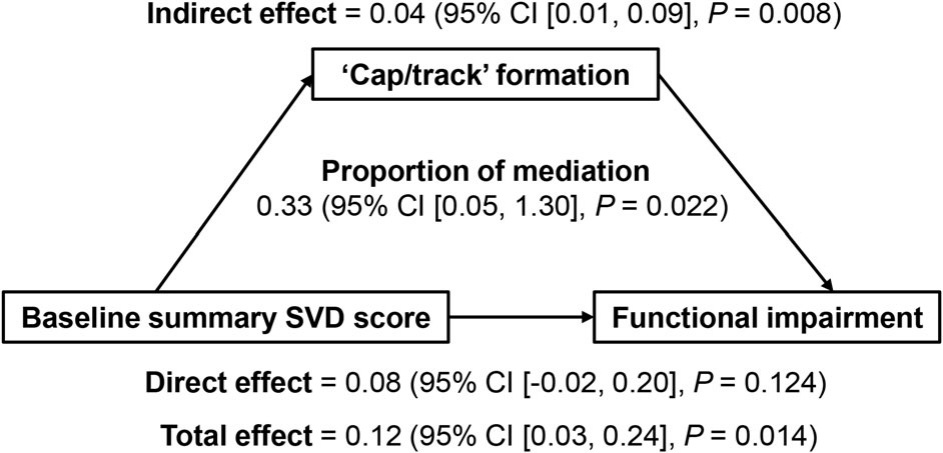
Illustration of the results of mediation analysis. The baseline summary small vessel disease (SVD) score is the exposure variable (X), the ‘cap/track’ formation is the mediator (M), and 1‐year functional impairment (modified Rankin Scale score ≥ 2) is the outcome variable (Y). Total effect = direct effect + indirect effect. The proportion of mediation effect is estimated by dividing the indirect effect by the total effect. The multivariable model included age, sex, National Institutes of Health Stroke Scale, index infarct diameter, and recurrent cerebrovascular event as covariates.

Given that SVD is associated with functional outcome after stroke,^31^ we performed mediation analysis using summary SVD score as the exposure, ‘cap/track’ formation as the mediator, and mRS ≥2 as the outcome, adjusting for age, sex, NIHSS, RSSI diameter, and recurrent cerebrovascular events. The results showed that ‘cap/track’ formation significantly mediated the effect of higher baseline SVD score on poorer functional outcome (indirect effect: 0.04, *p* = 0.008; direct effect: 0.08, *p* = 0.124; proportion of mediation effect: 33%, *p* = 0.022) (Fig 5).

## Discussion

In this longitudinal study of lacunar stroke patients with RSSI, we found that half developed ‘caps/tracks’ within 1 year after stroke. Larger RSSI diameter, RSSI located in white matter, higher SVD burden, and poorer white matter microstructural integrity indicated by higher NAWM‐MD, predicted ‘cap/track’ formation. At 1 year, development of ‘caps/tracks’ was associated with poorer functional outcome, worse mobility, and recurrent cerebrovascular events, but not with cognitive impairment. Additionally, ‘cap/track’ formation mediated the association between baseline summary SVD score and worse functional outcome.

The fate of RSSI has attracted interest for its potential prognostic implications.^2^ While most studies focus on morphological changes within the lesion, less is known about secondary damage to remote brain regions.^32^ ‘Caps/tracks’, representing newly arising hyperintense trajectories extending from the index RSSI, were first documented on MRI by Loos et al in 15 of 82 (18%) lacunar stroke patients during a 2‐year follow‐up.^8^ Subsequent research in Edinburgh lacunar stroke cohorts noted ‘caps/tracks’ in 42 of 79 (53%) over 1–5 years (partially overlapping with our current analysis).^9^ Our study builds upon the previous work, showing that 93 of 185 (50.3%) developed ‘caps/tracks’ within 1 year. In contrast, another study of basal ganglia RSSI reported a much lower incidence (8 of 78: 10%) over a median follow‐up of 304 days (range 19–856),^10^ aligning with our finding of RSSI in basal ganglia gray matter being less likely to form ‘caps/tracks’ than in white matter. Differences in study populations, MRI protocols, and follow‐up intervals likely explain the heterogeneity across studies.

The pathogenesis of ‘cap/track’ formation remains incompletely understood. Our findings suggest that ‘cap/track’ development depends on the extent of the primary lesion, indicated by larger RSSI diameter and its location relative to the white matter tracts. This phenomenon might be explained by anterograde (Wallerian), retrograde, or trans‐synaptic degeneration of interconnected fiber tracts, wherein distal axons undergo disintegration, demyelination, and gliosis following an injury to the neuronal cell body or proximal axon, with more fibers being affected by larger RSSI.^33^, ^34^, ^35^, ^36^, ^37^, ^38^ Interestingly, while most ‘caps/tracks’ follow the corticospinal tract (Fig 1), our study identified intra‐hemispheric and transcallosal inter‐hemispheric ‘tracks’ (Fig 2). Likewise, analysis in patients with CADASIL revealed that most WMH (linear in appearance, resembling a ‘track’) in the corpus callosum crossed a nearby lacune, possibly indicating a similar process.^39^ Furthermore, these observations are supported by animal research showing histological evidence of secondary callosal degeneration with axonal and myelin impairment following a primary focal striatal ischemia in rats.^40^, ^41^ Admittedly, we did not specifically assess remote cortical thinning which has been reported to reflect secondary changes after subcortical infarcts,^12^, ^14^ so our insights into the pathological processes underlying ‘caps/tracks’ are limited, necessitating further research.

In addition to RSSI size and location, we found that a higher baseline SVD burden and lower white matter microstructural integrity predict ‘cap/track’ formation. This implies that both macro‐ and microstructural damage related to SVD are potential pathological underpinnings of ‘caps/tracks’. Furthermore, the statistical finding of a mediation effect of ‘caps/tracks’ on the relationship between SVD and worse functional outcomes offers key mechanistic insights. It is plausible that existing SVD pathology may accelerate the conversion of normal white matter into ‘caps/tracks’, contributing to secondary damage. Further studies integrating neuroimaging with pathology data are needed to elucidate the underlying mechanism between SVD and ‘caps/tracks’.

We demonstrate several important clinical implications of ‘caps/tracks’. First, ‘cap/track’ formation after RSSI mainly affects physical functioning manifesting as disability, slower gait speed, and worse SIS physical domain score. This might reflect that symptomatic RSSI generally locates in critical motor and/or sensory tracts, whereas similarly small subcortical infarcts occurring outside the corticospinal tracts are less noticeable to patients.^42^, ^43^ It is therefore plausible that progression of damage in the tracts adjacent to the RSSI would result in worse physical function in the long term. Indeed, previous studies have shown that chronic small subcortical infarcts were associated with microstructural damage in the affected white matter tracts and linked to poorer motor recovery.^44^, ^45^ Second, a higher risk of ‘cap/track’ formation, and hence worse physical functional and mobility outcomes, can be predicted early after lacunar stroke by the larger RSSI at presentation and worse baseline SVD burden. This knowledge may help to identify high‐risk patients who would benefit most from personalized physical rehabilitation therapy and predict their prognosis. Third, the development of ‘caps/tracks’ indicates frailty of the brain, as evidenced by the more abnormal NAWM‐MD at baseline and higher rate of recurrent stroke and incident infarcts on MRI by 1 year in our study. This ‘vulnerability’ concept is consistent with evidence showing greater infarct growth after territorial stroke in acute stroke patients with worse SVD, that is, greater susceptibility to ischemia.^46^ Additionally, our findings of a higher rate of recurrent cerebrovascular events align with previous studies where DWI‐positive index stroke patients had worse baseline WMH and were more likely to have a new lesion on MRI at 1 year (symptomatic or asymptomatic) than the DWI‐negative index stroke patients.^16^ Taken together, ‘caps/tracks’ may serve as new markers of vulnerability to progressive tissue damage with potentially adverse clinical consequences. Identifying these markers may help develop therapies targeting secondary neurodegeneration, thereby enhancing brain resilience and improving management of lacunar stroke.

We also demonstrate important research implications, including for clinical trials. In addition to the impact of SVD burden on future risk of stroke and dementia,^31^, ^47^ we demonstrate its effects on functional outcome mediated by ‘cap/track’ formation. These results highlight the relevance of (1) adjusting for baseline SVD severity in observational studies of long‐term outcomes,^48^ and (2) minimizing on SVD burden in randomized controlled trials to avoid baseline imbalance in key prognostic variables.^49^ As shown here, ‘cap/track’ formation is quantifiable and might serve as an early or intermediate outcome marker of treatment efficacy in trials of novel interventions aiming to improve outcomes after lacunar stroke. Since many SVD features evolve slowly, trials in SVD using conventional imaging outcomes (eg, WMH progression) require long follow‐up periods.^50^ However, the median time to ‘cap/track’ detection was 198 days (IQR 135–354), with most appearing within 3–6 months after lacunar stroke. Thus, a therapeutic intervention aiming to improve functional outcome after lacunar stroke might begin to show efficacy within 6 months. Additionally, ‘cap/track’ formation is common enough (50% of patients) to power a trial at 90% with a relatively small sample size. Therefore, we suggest that ‘caps/tracks’ should be included, among other baseline prognostic factors in future trials of RSSI.

The strengths of this study include the longitudinal design with a well‐defined group of lacunar stroke patients, using standardized image acquisition protocol primarily on 3D sequences, conducting serial MRI on the same scanner for each individual, and image assessment by a team with expertise in SVD blinded to clinical data. We assessed physical and cognitive function both subjectively and objectively, as well as recurrent cerebrovascular events clinically and radiologically. This approach enabled a comprehensive analysis of the prognostic implications of ‘caps/tracks’.

Some limitations should be acknowledged. First, although this is one of the largest cohorts of lacunar stroke patients with pre‐specified imaging follow‐up, the sample size remains relatively small. Second, we combined data from 3 studies at the same center to increase statistical power, as participants were recruited similarly with comparable assessments and long‐term follow‐up. However, differences in MRI protocols (eg, field strength, acquisition parameters) could influence our results. Although the frequency of ‘cap/track’ detection was consistent across the cohorts, less frequent MRI in MSS‐2 cohort may limit the precision of our timing estimates for ‘cap/track’ development. Even with the improved scanning frequency in MSS‐3 cohort, some incident DWI‐positive lesions may still have been missed, which could weaken the association between ‘caps/tracks’ and recurrent cerebrovascular events. Further investigation into the effects of MRI acquisition parameters and scan intervals on ‘cap/track’ visibility and incident infarct detection is needed. Third, we enrolled only participants with minor stroke, defined as mRS ≤2 at recruitment, which could potentially underestimate the association between ‘caps/tracks’ and clinical outcomes. Fourth, the lack of association between ‘caps/tracks’ and cognitive impairment may be due to the short follow‐up period and the limited sensitivity of the MoCA/SIS to detect subtle changes in specific cognitive domains. Such associations may become apparent in our ongoing long‐term follow‐up at 3–5 years for the MSS‐3 study. Fifth, there are some missing data on TUG, SIS, and cognitive tests. Since elderly patients with poor clinical outcomes are less likely to attend follow‐up, this might have introduced attrition bias. Sixth, although we adjusted for known prognostic variables, the possibility of residual confounding cannot be excluded. Finally, our study was conducted only in the United Kingdom with relatively young participants, the generalizability of our results to other populations remains to be established. Further research is required to verify our findings in larger, multicenter, and ethnically diverse cohorts.

In conclusion, this study offers novel insights into the clinical relevance of ‘caps/tracks’ following RSSI. RSSI with ‘cap/track’ formation showed distinct characteristics, including specific infarct features, higher SVD burden and worse clinical outcomes at 1 year, highlighting their role as biomarkers of vulnerability to progressive SVD‐related brain injury. Assessing ‘caps/tracks’ on follow‐up MRI may aid prognostication and tailored management of lacunar stroke in clinical practice. Future trials should test ‘caps/tracks’ as intermediate biomarkers of treatment efficacy to improve outcome after lacunar stroke.

## Author Contributions

J.M.W., F.N.D., Y.C. contributed to the concept and design of the study. All authors contributed to acquisition and analysis of data. Y.C., C.A.R., U.C., D.J.G., M.V.H., M.S.S., F.M.C., X.L., A.C.C.J., S.M.M., C.M.J.L., S.D.J.M., and J.M.W. contributed to drafting the text or preparing the figures. A complete list of the MSS‐2, MSS‐3 and the INVESTIGATE‐SVDs Study Group collaborators is available in the Data S1.

## Potential Conflicts of Interest

Nothing to report.

## Supporting information


**Data S1.** Supporting Information.

## Data Availability

Anonymized data are available upon reasonable request to the chief investigator.
