# Supplementary material for: Clinical Relevance of ‘Cap’ and ‘Track’ Development after Recent Small Subcortical Infarct
Source: Ann Neurol. 2025 Jan 17;97(5):942–55. doi: 10.1002/ana.27182 (PMC12010063; doi:10.1002/ana.27182)
Supplement: Supplementary file 1 — Data S1. Supporting Information. [file ANA-97-942-s001.docx]

**SUPPLEMENTARY MATERIAL**

**Supplementary Methods**

**Propensity score weighting**

We employed inverse probability of treatment weighting (IPTW) estimation with propensity scores to balance the distribution of confounding variables between the group with ‘caps/tracks’ and those without.^1^ The propensity score is the probability that an individual is assigned to (or received) a specific treatment condition or exposure (i.e. presence or absence of ‘caps/tracks’ is the exposure in the current study) given a set of observed covariates. We estimated propensity score weights using generalized boosted modeling (GBM), a machine learning multivariate non-parametric regression technique that iteratively estimates the propensity score of individuals to maximize balance in observed covariates.^2^ GBM can effectively integrate interactions among multiple factors, hence reducing the risk of model misspecification.^3^ Previous studies have shown that GBM outperforms logistic regression in propensity score estimation.^2,4^

In establishing the propensity score with GBM model, we aimed to estimate the average treatment effect (ATE). We employed the mean Kolmogorov-Smirnov (KS.mean) statistic as a stopping criterion to assess and summarize balance across confounding variables. A 10,000-tree GBM model was utilized, with an interaction depth of 3 and a shrinkage value of 0.01. The final propensity scores for the two groups based on observable covariates were estimated using the R-package ‘twang’.^5^ Then, we conducted IPTW-adjusted regression analyses to compare clinical outcomes between participants who developed ‘caps/tracks’ and those did not. R-package ‘survey’ was used for the weighted regression analyses.

**Table S1. Baseline characteristics and outcomes of participants stratified by source study**

| **Characteristics^a^** | **MSS-2 study**  **(n=61)** | **MSS-3 study and INVESTIGATE-SVDs study^b^**  **(n=124)** |
| --- | --- | --- |
| **Demographics** |  |  |
| Age, year, mean (SD) | 65.3 (11.0) | 64.5 (11.3) |
| Male sex, n (%) | 39 (63.9) | 82 (66.1) |
| Education years, median [IQR] | 10 [10–12] | 11 [10–13] |
| **Risk factors** |  |  |
| Hypertension, n (%) | 47 (77.0) | 96 (77.4) |
| Diabetes mellitus, n (%) | 6 (9.8) | 32 (25.8) |
| Hyperlipidemia, n (%) | 39 (63.9) | 99 (79.8) |
| Current smoking, n (%) | 24 (39.3) | 32 (25.8) |
| Ischemic heart disease, n (%) | 6 (9.8) | 12 (9.7) |
| Atrial fibrillation, n (%) | 4 (6.6) | 9 (7.3) |
| History of stroke or TIA, n (%) | 11 (18.0) | 22 (17.7) |
| Vascular sum score, median [IQR] | 2 [1–2] | 2 [2–3] |
| **Baseline clinical data** |  |  |
| NIHSS, median [IQR] | 2 [2–4] | 1 [1–2] |
| MoCA, median [IQR] | 26 [23–29] | 25 [23–27] |
| NART, median [IQR] | 41 [33–45] | 35 [27–41] |
| Systolic blood pressure, mmHg, mean (SD) | 148.8 (25.6) | 151.4 (19.6) |
| Diastolic blood pressure, mmHg, mean (SD) | 83.4 (14.6) | 85.6 (12.8) |
| **Stroke onset to MRI time, days, median [IQR]** |  |  |
| Time to diagnostic scan | 4 [2–6] | 5 [3–15] |
| Time to baseline scan | 46 [36–65] | 55 [38–75) |
| Time to 1-year scan | 386 [366–415] | 429 [398–454] |
| **RSSI diameter at diagnosis, mm, median [IQR]** | 12.6 [9.2–15.0] | 12.5 [7.8–15.5] |
| **RSSI location, n (%)** |  |  |
| Lentiform nucleus | 5 (8.2) | 8 (6.5) |
| Internal or external capsule | 9 (14.8) | 26 (21.0) |
| Thalamus | 13 (21.3) | 30 (24.2) |
| Centrum semiovale | 28 (45.9) | 41 (33.1) |
| Brainstem | 6 (9.8) | 19 (15.3) |
| **Baseline SVD markers** |  |  |
| Presence of lacunes, n (%) | 26 (42.6) | 81 (65.3) |
| No. of lacunes, median [IQR] | 0 [0–1] | 2 [0–5] |
| Periventricular WMH Fazekas score, median [IQR] | 2 [1–2] | 2 [1–3] |
| Deep WMH Fazekas score, median [IQR] | 1 [1–2] | 1 [1–2] |
| WMH volume as percent ICV, median [IQR] | 1.2 [0.6–2.7] | 0.6 [0.3–1.4] |
| Moderate-to-severe BG-PVS, n (%) | 40 (65.6) | 85 (68.5) |
| Moderate-to-severe CSO-PVS, n (%) | 40 (65.6) | 99 (79.8) |
| Presence of cerebral microbleeds, n (%) | 18 (29.5) | 35 (28.2) |
| No. of cerebral microbleeds, median [IQR] | 0 [0–2] | 0 [0–1] |
| NAWM FA, median [IQR] | 0.26 [0.24–0.27] | 0.42 [0.41–0.44] |
| NAWM MD, 10^–3^ mm^2^/s, median [IQR] | 0.78 [0.76–0.81] | 0.76 [0.74–0.78] |
| Summary SVD score, median [IQR] | 2 (1–3) | 2 (1–3) |
| **1-year follow-up data** |  |  |
| Presence of ‘caps/tracks’, n (%) | 31 (50.8) | 62 (50.0) |
| mRS, median [IQR] | 1 [1–2] | 1 [0–1] |
| TUG, median [IQR] | 10.5 [8.6–11.8] | 10.6 [9.1–13.0] |
| SIS physical domain score, median [IQR] | 91.2 [73.5–98.1] | 92.3 [78.2–97.4] |
| MoCA, median [IQR] | 27.0 [23.0–29.0] | 26.0 [24.0–29.0] |
| SIS memory/thinking domain score, median [IQR] | 89.3 [71.4–100.0] | 89.3 [75.0–100.0] |
| Composite recurrent cerebrovascular event, n (%) | 10 (16.4) | 40 (32.3) |
| Recurrent stroke or TIA, n (%) | 6 (9.8) | 12 (9.7) |
| Incident infarct on MRI, n (%) | 7 (11.5) | 37 (29.8) |

^a^The number of participants with missing values was 16 for education years, 46 for baseline MoCA, 18 for NART, 6 for WMH volume, 13 for diffusion measures, 40 for TUG, 23 for SIS, 23 for 1-year MoCA.

^b^The MSS-3 study and INVESTIGATE-SVDs study were combined due to the small sample size of the INVESTIGATE-SVDs study (n=6) and the use of the same imaging protocol in both studies.

Abbreviations: BG-PVS = basal ganglia perivascular spaces; CSO-PVS = centrum semiovale perivascular spaces; FA = fractional anisotropy; ICV = intracranial volume; IQR = interquartile range; MD = mean diffusivity; MoCA = Montreal Cognitive Assessment; mRS = modified Rankin Scale; NART = National Adult Reading Test; NAWM = normal-appearing white matter; NIHSS = National Institutes of Health Stroke Scale; RSSI = recent small subcortical infarct; SD = standard deviation; SIS = Stroke Impact Scale; SVD = small vessel disease; TIA = transient ischemic attack; TUG = Timed Up-and-Go; WMH = white matter hyperintensities.

**Table S2. Baseline characteristics of participants with and without follow-up MRI**

| **Characteristics** | **Participants with follow-up**  **(n=185)** | **Participants without follow-up (n=31)** | ***P* value** |
| --- | --- | --- | --- |
| **Demographics** |  |  |  |
| Age, year, mean (SD) | 64.8 (11.2) | 69.1 (12.3) | 0.052 |
| Male sex, n (%) | 121 (65.4) | 21 (67.7) | 0.800 |
| **Risk factors** |  |  |  |
| Hypertension, n (%) | 143 (77.3) | 17 (54.8) | 0.008 |
| Diabetes, n (%) | 38 (20.5) | 7 (22.6) | 0.796 |
| Hyperlipidemia, n (%) | 138 (74.6) | 18 (58.1) | 0.057 |
| Current smoking, n (%) | 56 (30.3) | 10 (32.3) | 0.824 |
| Ischemic heart disease, n (%) | 18 (9.7) | 1 (3.2) | 0.237 |
| Atrial fibrillation, n (%) | 13 (7.0) | 2 (6.5) | 0.907 |
| History of stroke or TIA, n (%) | 33 (17.8) | 2 (6.5) | 0.111 |
| **Clinical data** |  |  |  |
| NIHSS, median (IQR) | 2 [1–3] | 2 [1–3] | 0.413 |
| Systolic blood pressure, mmHg, mean (SD) | 150.5 (21.8) | 155.3 (21.5) | 0.263 |
| Diastolic blood pressure, mmHg, mean (SD) | 84.9 (13.5) | 87.0 (11.0) | 0.414 |
| **Baseline SVD markers** |  |  |  |
| Presence of lacunes, n (%) | 107 (57.8) | 20 (64.5) | 0.484 |
| Periventricular WMH Fazekas score, median [IQR] | 2 [1–3] | 2 [1–3] | 0.652 |
| Deep WMH Fazekas score, median [IQR] | 1 [1–2] | 2 [1–2] | 0.345 |
| Moderate-to-severe BG-PVS, n (%) | 125 (67.6) | 19 (61.3) | 0.493 |
| Moderate-to-severe CSO-PVS, n (%) | 139 (75.1) | 26 (83.9) | 0.289 |
| Presence of cerebral microbleeds, n (%) | 53 (28.6) | 8 (25.8) | 0.745 |
| Summary SVD score, median [IQR] | 2 [1–3] | 2 [1–3] | 0.940 |

Abbreviations: BG-PVS = basal ganglia perivascular spaces; CSO-PVS = centrum semiovale perivascular spaces; IQR = interquartile range; NIHSS = National Institutes of Health Stroke Scale; SD = standard deviation; SVD = small vessel disease; TIA = transient ischemic attack; WMH = white matter hyperintensities.

**Table S3. Baseline characteristics and outcomes of participants stratified by ‘cap/track’ category**

| **Characteristics^a^** | **‘Cap/track’ absent**  **(n=92)** | **‘Cap’ or ‘track’ alone (n=54)** | **Both ‘cap’ and ‘track’ (n=39)** | ***P* value^b^** |
| --- | --- | --- | --- | --- |
| **Demographics** |  |  |  |  |
| Age, year, mean (SD) | 63.8 (12.0) | 69.1 (9.4) | 61.1 (9.5) | 0.001 |
| Male sex, n (%) | 62 (67.4) | 59 (63.4) | 25 (64.1) | 0.847 |
| Education years, median [IQR] | 11 [10–13] | 10 [10–12] | 11 [11–13] | 0.044 |
| **Risk factors** |  |  |  |  |
| Hypertension, n (%) | 69 (75.0) | 44 (81.5) | 30 (76.9) | 0.664 |
| Diabetes mellitus, n (%) | 17 (18.5) | 12 (22.2) | 9 (23.1) | 0.784 |
| Hyperlipidemia, n (%) | 72 (78.3) | 36 (66.7) | 30 (76.9) | 0.279 |
| Current smoking, n (%) | 28 (30.4) | 11 (20.4) | 17 (43.6) | 0.055 |
| Ischemic heart disease, n (%) | 9 (9.8) | 7 (13.0) | 2 (5.1) | 0.453 |
| Atrial fibrillation, n (%) | 6 (6.5) | 6 (11.1) | 1 (2.6) | 0.272 |
| History of stroke or TIA, n (%) | 16 (17.4) | 12 (22.2) | 5 (12.8) | 0.499 |
| Vascular sum score, median [IQR] | 2 [2–3] | 2 [2–2] | 2 [2–3] | 0.251 |
| **Baseline clinical data** |  |  |  |  |
| NIHSS, median [IQR] | 2 [1–2] | 2 [1–2] | 2 [1–4] | 0.202 |
| MoCA, median [IQR] | 24 [23–27] | 25 [23–27] | 26 [24–26] | 0.840 |
| NART, median [IQR] | 35 [28–42] | 38 [28–43] | 36 [31–40] | 0.692 |
| Systolic blood pressure, mmHg, mean (SD) | 148.8 (21.6) | 151.2 (22.4) | 153.7 (20.7) | 0.478 |
| Diastolic blood pressure, mmHg, mean (SD) | 84.6 (12.2) | 83.7 (13.7) | 87.0 (15.6) | 0.498 |
| **Stroke onset to MRI time, days, median (IQR)** |  |  |  |  |
| Time to diagnostic scan | 4 [2–9] | 4 [3–12] | 6 [3–15] | 0.193 |
| Time to baseline scan | 52 [36–70] | 53 [38–67] | 53 [38–78] | 0.712 |
| Time to 1-year scan | 419 [380–448] | 419 [385–447] | 406 [373–455] | 0.751 |
| **RSSI diameter at diagnosis, mm, median [IQR]** | 9.6 [6.0–14.2] | 13.7 [11.0–16.3] | 13.5 [11.0–16.5] | <0.001 |
| **RSSI location, n (%)** |  |  |  | <0.001 |
| Lentiform nucleus | 6 (6.5) | 3 (5.6) | 4 (10.3) |  |
| Internal or external capsule | 13 (14.1) | 11 (20.4) | 11 (28.2) |  |
| Thalamus | 37 (40.2) | 5 (9.3) | 1 (2.6) |  |
| Centrum semiovale | 20 (21.7) | 28 (51.9) | 21 (53.8) |  |
| Brainstem | 16 (17.4) | 7 (13.0) | 2 (5.1) |  |
| **Baseline SVD markers** |  |  |  |  |
| Presence of lacunes, n (%) | 47 (51.1) | 32 (59.3) | 28 (71.8) | 0.087 |
| No. of lacunes, median [IQR] | 1 [0–2] | 1 [0–4] | 2 [0–6] | 0.005 |
| Periventricular WMH Fazekas score, median [IQR] | 1 [1–2] | 2 [2–3] | 2 [1–3] | <0.001 |
| Deep WMH Fazekas score, median [IQR] | 1 [1–1] | 2 [1–2] | 2 [1–2] | <0.001 |
| WMH volume as percent ICV, median [IQR] | 0.5 [0.2–1.0] | 1.1 [0.6–2.4] | 1.4 [0.5–2.3] | <0.001 |
| Moderate-to-severe BG-PVS, n (%) | 52 (56.5) | 43 (79.6) | 30 (76.9) | 0.006 |
| Moderate-to-severe CSO-PVS, n (%) | 64 (69.6) | 44 (81.5) | 31 (79.5) | 0.214 |
| Presence of cerebral microbleeds, n (%) | 18 (19.6) | 22 (40.7) | 13 (33.3) | 0.018 |
| No. of cerebral microbleeds, median [IQR] | 0 [0–0] | 0 [0–2] | 0 [0–2] | 0.034 |
| NAWM FA, median [IQR] | 0.41 [0.28–0.43] | 0.40 [0.25–0.42] | 0.40 [0.37–0.43] | 0.167 |
| NAWM MD, 10^–3^ mm^2^/s, median [IQR] | 0.76 [0.74–0.78] | 0.77 [0.76–0.79] | 0.78 [0.76–0.80] | 0.023 |
| Summary SVD score, median [IQR] | 2 [1–2] | 3 [2–4] | 3 [2–3] | <0.001 |
| **1-year follow-up data** |  |  |  |  |
| mRS, median [IQR] | 1 [0–1] | 1 [0–2] | 1 [0–2] | 0.080 |
| mRS ≥ 2, n (%) | 13 (14.1) | 17 (31.5) | 18 (46.2) | <0.001 |
| TUG, median [IQR] | 10.0 [8.9–11.9] | 10.9 [9.0–16.0] | 11.4 [9.5–14.8] | 0.055 |
| SIS physical domain score, median [IQR] | 93.1 [83.6–98.1] | 94.1 [69.4–97.9] | 85.6 [52.4–95.9] | 0.084 |
| MoCA, median [IQR] | 26.0 [24.0–29.0] | 26.0 [24.0–29.0] | 27.0 [25.0–29.0] | 0.638 |
| SIS memory/thinking domain score, median [IQR] | 89.3 [75.0–100.0] | 89.3 [78.6–100.0] | 82.1 [57.1–100.0] | 0.555 |
| Composite recurrent cerebrovascular event, n (%) | 15 (16.3) | 20 (37.0) | 15 (38.5) | 0.005 |
| Recurrent stroke or TIA, n (%) | 6 (6.5) | 4 (7.4) | 8 (20.5) | 0.037 |
| Incident infarct on MRI, n (%) | 11 (12.0) | 19 (35.2) | 14 (35.9) | <0.001 |

^a^The number of participants with missing values was 16 for education years, 46 for baseline MoCA, 18 for NART, 6 for WMH volume, 13 for diffusion measures, 40 for TUG, 23 for SIS, 23 for 1-year MoCA.

^b^Categorical variables were analyzed using the χ^2^ or Fisher exact test, one-way ANOVA test was employed for continuous variables with a normal distribution, and the Kruskal-Wallis test was used for other continuous variables.

Abbreviations: BG-PVS = basal ganglia perivascular spaces; CSO-PVS = centrum semiovale perivascular spaces; FA = fractional anisotropy; ICV = intracranial volume; IQR = interquartile range; MD = mean diffusivity; MoCA = Montreal Cognitive Assessment; mRS = modified Rankin Scale; NART = National Adult Reading Test; NAWM = normal-appearing white matter; NIHSS = National Institutes of Health Stroke Scale; RSSI = recent small subcortical infarct; SD = standard deviation; SIS = Stroke Impact Scale; SVD = small vessel disease; TIA = transient ischemic attack; TUG = Timed Up-and-Go; WMH = white matter hyperintensities.

**Table S4. Locations of 104 incident infarcts in 44 of 185 participants**

|  | **Incident infarct total (n=104)** |
| --- | --- |
| **Incident small subcortical infarct locations, n (%)** |  |
| Centrum semiovale | 28 (26.9) |
| Juxta-cortical | 11 (10.6) |
| Optic radiation | 9 (8.6) |
| Internal border zone | 8 (7.7) |
| Pons | 8 (7.7) |
| Lentiform nucleus | 7 (6.7) |
| Thalamus | 5 (4.8) |
| Cerebellum | 5 (4.8) |
| Anterior temporal | 3 (2.9) |
| Internal capsule | 2 (1.9) |
| External capsule | 2 (1.9) |
| Splenium of corpus callosum | 2 (1.9) |
| Anterior frontal | 1 (1.0) |
| Caudate nucleus | 1 (1.0) |
| **Incident cortical infarct locations, n (%)** |  |
| Small middle cerebral artery territory | 5 (4.8) |
| Posterior borderzone | 3 (2.9) |
| <Half posterior cerebral artery territory | 1 (1.0) |
| Anterior borderzone | 1 (1.0) |
| <Half anterior cerebral artery territory | 1 (1.0) |
| Posterior half peripheral middle cerebral artery territory | 1 (1.0) |

**Table S5. Propensity score-weighted regression analyses of associations between ‘cap/track’ and outcomes**

|  | **Effect size (95% CI)^d^** | ***P* value** |
| --- | --- | --- |
| **Physical function^a^** |  |  |
| mRS ≥ 2 | OR: 3.68 (1.71–7.93) | 0.001 |
| TUG | β: 0.16 (0.04–0.29) | 0.008 |
| SIS physical domain score | β: -8.49 (-14.68 – -2.31) | 0.007 |
| **Cognition^b^** |  |  |
| MoCA < 26 | OR: 0.88 (0.45–1.74) | 0.713 |
| SIS memory/thinking domain score | β: -1.07 (-6.44–4.30) | 0.695 |
| **Recurrent cerebrovascular event^c^** |  |  |
| Composite outcome | HR: 2.26 (1.18–4.31) | 0.014 |
| Recurrent stroke or TIA | HR: 4.46 (1.64–12.13) | 0.003 |
| Incident infarct on MRI | OR: 3.86 (1.72–8.66) | 0.001 |

^a^Propensity score was estimated using the following covariates: age, sex, NIHSS, recurrent cerebrovascular event, RSSI diameter, RSSI location, number of lacunes, number of cerebral microbleeds, moderate-to-severe basal ganglia perivascular spaces, and total Fazekas score.

^b^Propensity score was estimated using the following covariates: age, sex, education year, NART, baseline MoCA, RSSI diameter, RSSI location, number of lacunes, number of cerebral microbleeds, moderate-to-severe basal ganglia perivascular spaces, and total Fazekas score.

^c^Propensity score was estimated using the following covariates: age, sex, vascular sum score, history of stroke/TIA, RSSI diameter, RSSI location, number of lacunes, number of cerebral microbleeds, moderate-to-severe basal ganglia perivascular spaces, and total Fazekas score.

^d^The effect size of OR was estimated using weighted logistic regression, the standardized beta coefficient (β) using weighted linear regression, and the HR using weighted Cox proportional hazards regression.

Abbreviations: CI = confidence interval; HR = hazard ratio; MoCA = Montreal Cognitive Assessment; mRS = modified Rankin Scale; NART = National Adult Reading Test; NIHSS = National Institutes of Health Stroke Scale; OR = odds ratio; RSSI = recent small subcortical infarct; SIS = Stroke Impact Scale; TIA = transient ischemic attack; TUG = Timed Up-and-Go.

**Table S6. MSS-2 and MSS-3 study collaborators**

| Name and Degree | Affiliation | Role |
| --- | --- | --- |
| Ian Marshall, PhD | Centre for Clinical Brain Sciences, UK Dementia Research Institute, University of Edinburgh, Edinburgh, UK | Clinical Fellow |
| Olivia K.L. Hamilton, PhD | MRC/CSO Social and Public Health Sciences Unit, School of Health and Wellbeing, University of Glasgow, Glasgow, UK | Data acquisition |
| Ellen Backhouse, PhD | Centre for Clinical Brain Sciences, UK Dementia Research Institute, University of Edinburgh, Edinburgh, UK | Data acquisition |
| Will Hewins, BSc, MSc | Centre for Clinical Brain Sciences, UK Dementia Research Institute, University of Edinburgh, Edinburgh, UK | Data acquisition |
| Rachel Locherty | Centre for Clinical Brain Sciences, UK Dementia Research Institute, University of Edinburgh, Edinburgh, UK | Data acquisition |
| Emilie Sleight, PhD | Centre for Clinical Brain Sciences, UK Dementia Research Institute, University of Edinburgh, Edinburgh, UK | MRI vascular function acquisition/analysis |
| Alasdair G. Morgan, PhD | Centre for Clinical Brain Sciences, UK Dementia Research Institute, University of Edinburgh, Edinburgh, UK | MRI vascular function acquisition/analysis |
| Cameron Manning, PhD | Centre for Clinical Brain Sciences, UK Dementia Research Institute, University of Edinburgh, Edinburgh, UK | MRI vascular function acquisition/analysis |
| Iona Hamilton | Edinburgh Imaging Facility (Royal Infirmary of Edinburgh), University of Edinburgh, Edinburgh, UK | Radiographer |
| Gayle Barclay | Edinburgh Imaging Facility (Royal Infirmary of Edinburgh), University of Edinburgh, Edinburgh, UK | Radiographer |
| Donna McIntyre | Edinburgh Imaging Facility (Royal Infirmary of Edinburgh), University of Edinburgh, Edinburgh, UK | Radiographer |
| Charlotte Jardine | Edinburgh Imaging Facility (Royal Infirmary of Edinburgh), University of Edinburgh, Edinburgh, UK | Radiographer |
| Dominic Job, PhD | Centre for Clinical Brain Sciences, UK Dementia Research Institute, University of Edinburgh, Edinburgh, UK | Programming/data management |
| David Perry, PhD | Centre for Clinical Brain Sciences, UK Dementia Research Institute, University of Edinburgh, Edinburgh, UK | Programming/data management |
| Tom MacGillivray, PgCAP | Centre for Clinical Brain Sciences, UK Dementia Research Institute, University of Edinburgh, Edinburgh, UK | Data acquisition |
| Charlene Hamid | Centre for Clinical Brain Sciences, UK Dementia Research Institute, University of Edinburgh, Edinburgh, UK | Data acquisition |

**Table S7. INVESTIGATE-SVDs study collaborators, Part of the SVDs@Target project**

| Name and Degree | Affiliation | Role |
| --- | --- | --- |
| Martin Dichgans, MD | Institute for Stroke and Dementia Research (ISD), University Hospital, LMU Munich, 81377 Munich, Germany | PI |
| Anna Kopczak, MD | Institute for Stroke and Dementia Research (ISD), University Hospital, LMU Munich, 81377 Munich, Germany | Investigator |
| Marco Duering, MD | Institute for Stroke and Dementia Research (ISD), University Hospital, LMU Munich, 81377 Munich, Germany | Investigator |
| Benno Gesierich, PhD | Institute for Stroke and Dementia Research (ISD), University Hospital, LMU Munich, 81377 Munich, Germany | Investigator |
| Karin Waegemann, PhD | Institute for Stroke and Dementia Research (ISD), University Hospital, LMU Munich, 81377 Munich, Germany | Investigator |
| Michael Ingrisch, PhD | Department of Clinical Radiology, LMU-University of Munich, 81377 Munich, Germany | Investigator |
| Salvatore Rudilosso, PhD | Comprehensive Stroke Centre, Hospital Clinic, University of Barcelona, Spain | Investigator |
| Ernest Chui, BSc | Medical School, University of Edinburgh, Edinburgh | Student |
| Esther Janssen | Radboudumc, Donders Institute for Brain, Cognition and Behaviour | Student |
| Robert J van Oostenbrugge, MD | Department of Neurology, Maastricht University Medical Center, the Netherlands | PI |
| Julie Staals, MD | Department of Neurology, Maastricht University Medical Center, the Netherlands | Investigator |
| Maud van Dinther, MSc | Department of Neurology, Maastricht University Medical Center, the Netherlands | Investigator |
| Danielle Kerkhofs, MB ChB | Department of Neurology, Maastricht University Medical Center, the Netherlands | Investigator |
| Walter H Backes, PhD | Department of Medical Physics, Maastricht University Medical Center, Maastricht, The Netherlands | Investigator |
| Geert Jan Biessels, MD | Department of Neurology, UMC Utrecht Brain Center, University Medical Center Utrecht, the Netherlands | PI |
| Hilde van den Brink, MSc | Department of Neurology and Neurosurgery, UMC Utrecht Brain Center, University Medical Center Utrecht, Utrecht, The Netherlands | Investigator |
| Laurien P Onkenhout, MSc | Department of Neurology and Neurosurgery, UMC Utrecht Brain Center, University Medical Center Utrecht, Utrecht, The Netherlands | Investigator |
| Tine Arts, PhD | Department of Imaging Sciences, University Medical Center Utrecht, Utrecht, The Netherlands | Investigator |
| Stanley DT Pham, PhD | Department of Imaging Sciences, University Medical Center Utrecht, Utrecht, The Netherlands | Investigator |
| Jeroen Hendrikse, MD | Department of Imaging Sciences, University Medical Center Utrecht, Utrecht, The Netherlands | Investigator |
| Jaco JMZ Zwanenburg, PhD | Department of Imaging Sciences, University Medical Center Utrecht, Utrecht, The Netherlands | Investigator |
| Jeroen CW Siero, PhD | Department of Imaging Sciences, University Medical Center Utrecht, Utrecht, The Netherlands | Investigator |
| Alastair JS Webb, MD | Department of Clinical Neurosciences, Wolfson Centre for Prevention of Stroke and Dementia, University of Oxford, UK | PI |

**References**

1. Kurth T, Walker AM, Glynn RJ, et al. Results of multivariable logistic regression, propensity matching, propensity adjustment, and propensity-based weighting under conditions of nonuniform effect. Am J Epidemiol 2006;163:262–270.

2. McCaffrey DF, Griffin BA, Almirall D, et al. A tutorial on propensity score estimation for multiple treatments using generalized boosted models. Stat Med 2013;32:3388–3414.

3. McCaffrey DF, Ridgeway G, Morral AR. Propensity score estimation with boosted regression for evaluating causal effects in observational studies. Psychol Methods 2004;9:403–425.

4. Lee BK, Lessler J, Stuart EA. Improving propensity score weighting using machine learning. Stat Med 2010;29:337–346.

5. Salim HA, Pulli B, Yedavalli V, et al. Endovascular therapy versus medical management in isolated posterior cerebral artery acute ischemic stroke: A multinational multicenter propensity score-weighted study. Eur Stroke J 2024;23969873241291465.
